# Unraveling the interconversion pharmacokinetics and oral bioavailability of the major ginger constituents: [6]-gingerol, [6]-shogaol, and zingerone after single-dose administration in rats

**DOI:** 10.3389/fphar.2024.1391019

**Published:** 2024-06-06

**Authors:** Phanit Songvut, Watanyoo Nakareangrit, Wanida Cholpraipimolrat, Jackapun Kwangjai, Luksamee Worasuttayangkurn, Piyajit Watcharasit, Jutamaad Satayavivad

**Affiliations:** ^1^ Laboratory of Pharmacology, Chulabhorn Research Institute, Bangkok, Thailand; ^2^ Translational Research Unit, Chulabhorn Research Institute, Bangkok, Thailand; ^3^ Food and Drug Quality Unit, Chulabhorn Research Institute, Bangkok, Thailand; ^4^ Center of Excellence on Environmental Health and Toxicology (EHT), OPS, MHESI, Bangkok, Thailand

**Keywords:** [6]-gingerol, [6]-shogaol, zingerone, interconversion, pharmacokinetics, bioavailability

## Abstract

**Background:**

The available *in vitro* evidences suggest the inherent instability and interconvertibility of [6]-gingerol and [6]-shogaol. However, limited data on their *in vivo* interconversion hinder understanding of their influence on the pharmacokinetic profiles.

**Purpose:**

This study presents the first comprehensive *in vivo* investigation aiming to determine the interconversion pharmacokinetics in rats, and elucidate the oral bioavailability, target distribution, biotransformation, and excretion profiles of the key ginger constituents, [6]-gingerol, [6]-shogaol, and zingerone.

**Methods:**

The pharmacokinetics was investigated through single intravenous (3 mg/kg) or oral (30 mg/kg) administration of [6]-gingerol, [6]-shogaol, or zingerone, followed by the determination of their tissue distribution after oral dosing (30 mg/kg). Intravenous pharmacokinetics was leveraged to evaluate the interconversion, circumventing potential confounders associated with the oral route.

**Results:**

All rats tolerated these compounds throughout the pharmacokinetic study. The parent compounds exhibited rapid but partial absorption, and extensive organ distribution with substantial biotransformation, thereby limiting the oral bioavailability of each compound to below 2% when administered as pure compounds. Conversion of [6]-gingerol to [6]-shogaol after intravenous administration, demonstrated a significantly larger clearance compared to the reverse conversion ([6]-shogaol to [6]-gingerol). The irreversible metabolic clearance for both compounds was significantly greater than their reversible bioconversions. Furthermore, [6]-gingerol underwent biotransformation to zingerone. Conjugated glucuronides were eliminated partly through renal excretion, with minimal fecal excretion.

**Conclusion:**

This *in vivo* investigation demonstrates the influence of interconversion on the disposition kinetics of [6]-gingerol, [6]-shogaol, and zingerone, as evidenced by the findings in the systemic circulation. The study further highlights the importance of considering this interconversion and tissue distribution when determining the administration dosage of ginger constituent combinations for therapeutic benefits and clinical applications.

## 1 Introduction

The bioactive phenolic compounds, gingerol and shogaol, which are naturally present in ginger (*Zingiber officinale* Roscoe), have attracted research attention for their potential role in alleviating the symptoms of gastrointestinal (GI) disorders, particularly nausea and vomiting (Nikkhah Bodagh et al., 2019), and in promoting gastroprotective effects (Haniadka et al., 2013). The well-documented anti-inflammatory and antioxidant properties of these compounds (Mao et al., 2019), along with their potential activities in an *in silico* model (Zammel et al., 2021), warrant further clinical investigation into their therapeutic applications. Recent evidence also suggests their potential to mitigate age-related neurological disorders including Alzheimer’s disease, dementia, Parkinson’s disease, and stroke, through their emerging neuroprotective and neuropharmacological effects (Ha et al., 2012; Choi et al., 2018; Mohd Sahardi and Makpol, 2019; Talebi et al., 2021).

Among ginger’s bioactive constituents, [6]-gingerol (6G) and [6]-shogaol (6S) are present in significantly higher concentrations, contributing substantially to their therapeutic potential as major active compounds (Mukjerjee and Karati, 2022). Structurally, 6G possesses a *β*-hydroxy ketone moiety, with the hydroxy group positioned at the sixth carbon atom from the end of the side chain. In contrast, 6S possesses an *α*,*β*-unsaturated ketone, formed through the dehydration of 6G. Due to the inherent molecular instability of 6G and 6S, these two compounds show unpredictable pharmacokinetic behavior and variable pharmacological outcomes, restricting their clinical applications (Ding et al., 1991; Li et al., 2019; Zhang et al., 2022). This limitation necessitates further investigation into their pharmacokinetic alteration, including the reversible and irreversible bioconversion between these two key ginger components. An *in vitro* study using simulated gastric fluid suggests a reversible, first-order dehydration and hydration process for 6G and 6S, indicating their interconversion within the simulated digestive environment (Bhattarai et al., 2001; Bhattarai et al., 2007). However, the full extent of their *in vivo* pharmacokinetic behavior, particularly their interconversion processes, remains unexplored. Unlike the simplified conditions of *in vitro* models, the intricate interactions of digestive enzymes are present exclusively in an *in vivo* study, significantly influencing the conversion of these compounds. Gastrointestinal complex enzymes are absent in an *in vitro* setting, hindering the understanding of their impact on the bioavailability and disposition kinetics of 6G and 6S. Therefore, this preclinical study aims to investigate the interconversion pharmacokinetics in a rat model, addressing this limitation and enhancing our understanding of the *in vivo* interconversion of these promising bioactive compounds.

Given the potential impact of *in vivo* interconversion on the distribution and biotransformation processes, the current study also examined these aspects, particularly focusing on tissue distribution and on phase II glucuronide conjugation, a major metabolic pathway of these compounds supported by previous research (Wu et al., 2015; Wang et al., 2017; Li et al., 2019). Additionally, as 6G and 6S share a common molecular backbone with zingerone, a well-studied component of ginger known for its potent biological properties (Ahmad et al., 2015), this present study also investigated the potentially irreversible biotransformation of 6G and 6S into zingerone. The investigation was extended to determine the pharmacokinetics of zingerone and its glucuronide metabolite, providing a more comprehensive understanding of all related bioactive compounds of ginger (6G, 6S, and zingerone). Although previous evidence indicated restricted oral bioavailability for ginger constituents (Li et al., 2019; Zhang et al., 2022), most studies rely on ginger extract. The extract’s complexity hinders observation of the bioavailability of individual compounds. This study, therefore, investigated the oral bioavailability of each major bioactive compound through the administration of 6G, 6S, and zingerone.

Understanding the *in vivo* interconversion pharmacokinetics of major ginger constituents, will support the development of ginger-based therapeutic applications. Elucidating the tissue distribution of these compounds further enhances the potential for selective organ targeting.

## 2 Materials and methods

### 2.1 Chemicals

#### 2.1.1 Analytical standards

Analytical standards of [6]-gingerol (purity 100.0%), [6]-shogaol (purity 98.8%), zingerone (purity 99.5%), and internal standard (IS) of dihydrocapsaicin (purity 94.0%) were purchased from Sigma-Aldrich (St. Louis, MO, USA). Methanol (HPLC grade), acetonitrile (HPLC grade), and formic acid (AR grade) were obtained from Merck (Darmstadt, FR, Germany). Milli-Q water was obtained from a purification system (Millipore, Bedford, MA, USA), and was used throughout the analysis.

A lyophilized powder of *β*-glucuronidase type IX-A, derived from *Escherichia coli*, was purchased from Sigma-Aldrich (St. Louis, MO, USA). The powder had a glucuronidase activity ranging between 1,000,000 and 5,000,000 units/g of protein (glucuronidase activity = 2,354,185 units/g protein). Sodium dihydrogen phosphate and disodium hydrogen phosphate were also obtained from Sigma-Aldrich (St. Louis, MO, USA).

#### 2.1.2 Substances tested in animals

The pure substances tested in animals, [6]-gingerol (purity 99.0%) and [6]-shogaol (purity 97.1%), were purchased from Tauto Biotech (Shanghai, China). Zingerone (purity 99.5%) was purchased from Sigma-Aldrich (St. Louis, MO, USA). Dimethyl sulfoxide (DMSO) was obtained from Merck (Darmstadt, FR, Germany).

#### 2.1.3 Preparation of test solution

The test compounds were dissolved in a sterile vehicle for injection using dimethyl sulfoxide (DMSO) as a co-solvent to achieve a fully dissolved test drug solution suitable for intravenous injection. Briefly, a 6 mg/mL solution for i.v. administration was prepared by dissolving the compound in 200 µL of DMSO. This solution underwent vortex mixing for approximately 2 min before the addition of 800 µL of sterile water for injection to achieve a final volume of 1,000 µL (1 mL). Subsequently, the solution was filtered through a 0.2 µm polyvinylidene fluoride (PVDF) membrane (Chrome Tech, MN, USA) prior to i.v. injection. This solution was administered to rats at a volume of less than 300 µL/rat, calculated based on half of their body weight on the day of administration to achieve a receiving dose of 3 mg/kg for i.v. administration. Therefore, the amount of DMSO entering the bloodstream is calculated to be less than 0.1 mL/kg, which was considered safe based on the guideline and the previous literature involving intravenous administration of DMSO (Gad et al., 2006; Auletta, C.S., 2014; Thackaberry et al., 2014). The test compounds for p.o. administration were prepared using the same method.

### 2.2 Ethics and animal welfare

The animal use protocol was approved by the Institutional Animal Care and Use Committee (IACUC) of the Chulabhorn Research Institute, with the approval number PN 2022-01 (approval date: 28 February 2022). The animal study was conducted at the Laboratory Animal Center, Chulabhorn Research Institute (Bangkok, Thailand), under the standards of the American Association for Accreditation of Laboratory Animal Care (AAALAC).

### 2.3 Animals

Female Sprague Dawley rats were obtained from Nomura Siam International Co., Ltd. (Bangkok, Thailand). Female Sprague Dawley rats were selected for this study to investigate the potential tissue distribution of the test compounds to the ovaries. The animals were housed in the experimental facilities until they reached 15 weeks of age. In the pharmacokinetics study, adult rats with a body weight exceeding 350 g were included. They were accommodated in polysulfone shoebox cages (2 rats/cage) and were maintained under a 12-h light-dark cycle. Additionally, the rats were provided with *ad libitum* access to food and water.

### 2.4 Dose selection and justification for pharmacokinetic study

The dose selection for the intravenous and oral pharmacokinetic studies was based on previous pharmacokinetic investigations. Ding et al. (1991) used intravenous administration of 6-gingerol at a dosage of 3 mg/kg in rats. Oral dosage in the current study was determined based on a previous study examining ginger extract, in which the pharmacokinetics were evaluated following oral gavage of the extract with calculated doses ranging from 10.9 to 42.7 mg/kg of 6-gingerol, 6-shogaol, or zingerone in rats (Li et al., 2019).

### 2.5 Pharmacokinetic and tissue distribution studies

This study is composed of two sections; the first section involves an investigation of the interconversion pharmacokinetics of the pure compounds, and the second focuses on tissue distribution. All rats were transferred to metabolic cages and fasted overnight for at least 10 h. The animals were then randomly divided into four groups (6 rats/group) to receive a single intravenous (i.v.) administration of either 6G, 6S, zingerone, or vehicle via the lateral tail vein at a dosage of 3 mg/kg. After a 7-week washout period, the rats received 30 mg/kg of the same test compounds through a single oral (p.o.) administration. Following each administration, blood samples (300 µL) were collected in heparinized tubes from the lateral tail vein (while rats were under anesthesia with isoflurane) at specified time points: 0.083, 0.25, 0.5, 1, 1.5, 2, 3, 4, 6, 8, and 24 h after administration. An additional 1 mL of blood sample was collected from each rat at pre-dose (0 h) and post-dose (24 h) for clinical laboratory testing and safety evaluation. All heparinized blood samples were initially centrifuged at 5,000 × *g*, 4 °C, for 10 min. Subsequently, the plasma was carefully transferred to a cryotube as an aliquot sample and then stored at −40 °C until analysis. Urine and feces were collected during the intervals of 0–24 h and 24–48 h post-dosing, and their weights and volumes were documented. All collected urine and feces samples were stored at −40 °C until analysis.

Tissue distribution analysis was carried out after a 7-week washout period following the completion of the pharmacokinetic study. The animals received a single oral administration of either 6G, 6S, zingerone, or vehicle at a dosage of 30 mg/kg. Euthanasia was performed using an overdose of isoflurane (>10%) in an induction chamber, either one or 2 h after oral gavage (3 rats/timepoint), and death was confirmed by exsanguination. Subsequently, the stomach, small intestine, liver, large intestine, kidneys, heart, lung, hippocampus, cortex, spleen, ovaries, and spinal cord were collected. The dissected organs were thoroughly cleaned with a cold normal saline solution and the connective tissue was removed. The organs were then precisely weighed before being stored at −40 °C. All processes during tissue collection were performed on ice.

### 2.6 Safety evaluation

Plasma samples were collected at baseline (T_0_) and T_24 h_ for the determination of blood chemistry, including levels of blood urea nitrogen (BUN), creatinine, aspartate transaminase (AST), alanine transaminase (ALT), and alkaline phosphatase (ALP). The analyses were conducted by the Exclusive Veterinary Professional Laboratory Center (Bangkok, Thailand). The creatinine level was determined using an enzymatic oxidase assay, while AST and ALT levels were determined using the procedure of the International Federation of Clinical Chemistry (IFCC) without pyridoxal phosphate activation.

### 2.7 Sample preparation

Plasma samples were processed through protein precipitation. In brief, 50 µL of plasma was mixed with 200 µL of methanol containing 50 ng/mL of IS (dihydrocapsaicin). The mixtures were vortexed and centrifuged at 14,000 × *g* at 4 °C for 10 min. The supernatant was filtered through a 0.2 µM PVDF membrane (Chrome Tech, MN, USA) before analysis.

Urine samples were centrifuged, and 50 µL of urine was extracted with 200 µL of 100% methanol. The mixture was vortexed for 10 min and subsequently centrifuged at 14,000 × *g* at 4 °C for 10 min. Feces and organs were weighed and homogenized with methanol (50:50% w/v). The supernatants of the extracted urine, feces, and organs were also filtered through a 0.2 µM PVDF membrane (Chrome Tech, MN, USA) before analysis.

Investigation of glucuronide conjugations followed a previously published method with some modification (Wang et al., 2017; Songvut et al., 2023). A 50 μL aliquot of plasma, urine, or feces sample was incubated at 37 °C for a minimum of 30 min with 50 μL of *β*-glucuronidase (3,000 U/mL) in a sodium phosphate buffer (pH 6.8). For sample extraction, 150 μL of methanol containing 50 μg/L of IS was pipetted into the pre-incubated samples. Subsequently, the mixtures were vortexed for 10 min and then centrifuged at 14,000 × *g* at 4 °C for 10 min. The supernatants were filtered through a 0.2 µM PVDF membrane (Chrome Tech, MN, United States) and then analyzed using the validated LC-MS/MS method.

### 2.8 Analytical procedures and method validation

Quantitative analysis was conducted using liquid chromatography-tandem mass spectrometry (LC-MS/MS) with a Nexera X2 LCMS-8060NX instrument (Shimadzu, Japan). The instrument included a CBM-40lite system controller, CTO-40C column oven, SIL-40C XR autosampler, LC-40D XR solvent pump unit, FCV-DGU-403 degasser, and 20AH2 switching valve. The LC-MS/MS system was operated with a binary pump and equipped with a triple quadrupole mass spectrometer.

The LC system was run on a VertiSep AQS C18 column (100 mm × 3.0 mm, 3 μm) as a stationary phase, and it was protected by a VertiSep guard cartridge (3.0 × 10 mm) in a guard holder with a coupler (ID 2.1–7.8 mm). A pretreated sample of 10 µL was injected into a column maintained at a constant temperature of 40 °C. The flow rate of the mobile phase during the runtime analysis remained at 0.4 mL/min over 12 min. The chromatographic separation was performed using a gradient mobile phase consisting of 0.1% formic acid (A) and acetonitrile (B). The gradient profile was as follows: from 0 to 3.0 min, the mobile phase composition started at 60% v/v (B), followed by an increase to 90% v/v (B) between 3.0 and 8.0 min, maintaining at this concentration from 8.0 to 10.0 min. The gradient was then equilibrated to 60% v/v (B) from 10.0 to 12.0 min.

The analytical standards were individually dissolved in 100% methanol to optimize the collision energy (CE) and voltage for the MS-specific conditions of each compound. The MS parameters for the electrospray ionization (ESI) source in positive mode, operating in multiple reaction monitoring (MRM), were set as follows: heating gas flow = 15 L/min, nebulizer gas flow = 3 L/min, interface temperature = 400 °C, DL temperature = 250 °C, heat block temperature = 400 °C, and drying gas flow = 3 L/min. Transitions of mass/charge (*m/*z) were achieved for the precursor/product ion as follows: 277.05/177.10, 277.10/137.05, 177.15/117.15, and 308.10/137.15 for 6G, 6S, zingerone, and dihydrocapsaicin (IS), respectively (Supplementary Figures S1, S2). Data acquisition was performed using LabSolutions LCMS software, version 5.99 ep2 (Shimadzu, Japan).

#### 2.8.1 Stock standards and working solutions

The primary stock standard solutions of 6G, 6S, and zingerone were prepared at a concentration of 1 mg/mL. Each standard was accurately weighed and dissolved in 100% methanol (HPLC grade). A working solution of mixed standards was generated through serial dilution from the primary stock standard.

#### 2.8.2 Method validation

The LC-MS/MS analyses were performed based on a previously published method (Zhang et al., 2022) and were validated in accordance with the International Conference on Harmonization (ICH) guideline M10 on bioanalytical method validation (Guideline, 2022). The validation result indicated that all key parameters for this bioanalytical method met the required acceptance criteria, as presented in the Supplementary Tables S1, S2. The method demonstrated selectivity in blank samples, as no interference was detected at the retention times corresponding to the target compounds and IS. The calibration curves exhibited suitable linearity, with correlation coefficients (*R* ^2^) ≥ 0.99, over the concentration ranges of 3.91–2,000.00 μg/L with the lower limit of quantification (LLOQ) at 3.91 μg/L. The intra-day accuracy and inter-day accuracy were assessed using %RSD, while precision was evaluated through %CV. The corresponding validation parameters for each compound are summarized in Supplementary Tables S1, S2. The obtained results demonstrated that the %RSD values were within the acceptable range of 85%–115%, and %CVs met the criteria of ≤15%. The stability was assessed under freeze-thaw conditions, long-term conditions at −40 °C for 2 months, and in an autosampler at 4 °C for 24 h. The quality control (QC) samples were found to be stable within the accepted range of 85%–115% (Supplementary Table S2).

### 2.9 Pharmacokinetic analysis

Pharmacokinetic parameters were assessed using PK Solutions software (version 2.0) with non-compartmental analysis. The AUC was calculated using the Linear-Log Trapezoidal Method. The maximum plasma concentration (C_max_) and time to reach maximum plasma concentration (T_max_) were taken directly from the graph.

Plasma concentration-time profiles were plotted using GraphPad Prism 9.3.0 (GraphPad Software, USA). The absolute oral bioavailability was calculated as follows: F = (AUC p.o./dose p.o.)/(AUC i.v./dose i.v.). The percentage recovery of each administered compound was calculated as the total amount of the drug found in urine or feces divided by the administered dose. The tissue-to-plasma concentration ratios (*K*_p_) were calculated by dividing the concentration in the tissue by the corresponding plasma concentration at the respective sampling time point.

The interconversion between 6G and 6S was determined using previously published methods (Ebling and Jusko, 1986; Prueksaritanont et al., 2005). The clearance of 6G biotransformed into 6S was calculated as CL_12_, while the reverse clearance (6S → 6G) was calculated as CL_21_. The irreversible elimination clearances of 6G and 6S were calculated as CL_10_ and CL_20_, respectively. The following equations were used in accordance with the notation provided in Supplementary Table S3.

$${\text{CL}}_{12}=\frac{{\text{Dose}}^{\text{shogaol}}\;x{\text{AUC}}_{\text{shogaol}}^{\text{gingerol}}}{\left[\left({\text{AUC}}_{\text{gingerol}}^{\text{gingerol}}\;{x\;\text{AUC}}_{\text{shogaol}}^{\text{shogaol}}\right)-\;\left({\text{AUC}}_{\text{shogaol}}^{\text{gingerol}}\;{x\;\text{AUC}}_{\text{gingerol}}^{\text{shogaol}}\right)\right]}$$

$${\text{CL}}_{21}=\frac{{\text{Dose}}^{\text{gingerol}}\;x{\text{AUC}}_{\text{gingerol}}^{\text{shogaol}}}{\left[\left({\text{AUC}}_{\text{gingerol}}^{\text{gingerol}}\;{x\;\text{AUC}}_{\text{shogaol}}^{\text{shogaol}}\right)-\;\left({\text{AUC}}_{\text{shogaol}}^{\text{gingerol}}\;{x\;\text{AUC}}_{\text{gingerol}}^{\text{shogaol}}\right)\right]}$$

$${\text{CL}}_{10}=\frac{\left[\left({\text{Dose}}^{\text{gingerol}}\;{x\;\text{AUC}}_{\text{shogaol}}^{\text{shogaol}}\right)-\;\left({\text{Dose}}^{\text{shogaol}}\;{x\;\text{AUC}}_{\text{shogaol}}^{\text{gingerol}}\right)\right]}{\left[\left({\text{AUC}}_{\text{gingerol}}^{\text{gingerol}}\;{x\;\text{AUC}}_{\text{shogaol}}^{\text{shogaol}}\right)-\;\left({\text{AUC}}_{\text{shogaol}}^{\text{gingerol}}\;{x\;\text{AUC}}_{\text{gingerol}}^{\text{shogaol}}\right)\right]}$$

$${\text{CL}}_{20}=\frac{\left[\left({\text{Dose}}^{\text{shogaol}}\;{x\;\text{AUC}}_{\text{gingerol}}^{\text{gingerol}}\right)-\;\left({\text{Dose}}^{\text{gingerol}}\;{x\;\text{AUC}}_{\text{gingerol}}^{\text{shogaol}}\right)\right]}{\left[\left({\text{AUC}}_{\text{gingerol}}^{\text{gingerol}}\;{x\;\text{AUC}}_{\text{shogaol}}^{\text{shogaol}}\right)-\;\left({\text{AUC}}_{\text{shogaol}}^{\text{gingerol}}\;{x\;\text{AUC}}_{\text{gingerol}}^{\text{shogaol}}\right)\right]}$$

### 2.10 Statistical analysis

The data were analyzed using SPSS statistical software (Version 22.0). Continuous data are expressed as the mean ± standard deviation (SD), while non-continuous data are presented as the median [IQR]. The normality of the data was assessed using the Shapiro-Wilk test. To compare pharmacokinetic parameters between treatment groups receiving different compounds, analysis of variance (ANOVA) with Scheffe’s *post hoc* test was used for parametric analysis, and the Kruskal–Wallis test was applied for nonparametric analysis, as appropriate. Statistical significance was considered at *p*-values less than 0.05. For safety evaluation, biochemical markers of liver and kidney functions were analyzed using Student’s t-test to determine the significance between the tested group and the control group.

## 3 Results

### 3.1 Safety and tolerability

All rats exhibited tolerance to 6G, 6S, and zingerone (Table 1). No significant physiological changes or abnormalities in physical appearance were observed in any of the groups after the treatment. Levels of aspartate transaminase (AST) and alkaline phosphatase (ALP) were compared between each tested group and the control group to assess potential alterations in liver functions; no significant differences in AST and ALP levels were observed between the groups. A decrease in alanine transaminase (ALT) levels was observed during 24 h after the intravenous administration of 6G or 6S, as compared to the ALT levels of the control group. Creatinine levels, which were compared to assess potential changes in kidney functions, did not show any significant differences after the administration of the tested compounds. A statistically significant decrease in blood urea nitrogen (BUN) was observed after administering an oral zingerone when compared to the control group. These changes, however, were transient, remaining within the normal ranges for adult rats.

**TABLE 1:** Tolerability and safety profile after a single intravenous or oral administration of [6]-gingerol, [6]-shogaol and zingerone in rats.

Biochemical Parameters		Control		[6]-gingerol		[6]-shogaol		Zingerone	
		i.v	p.o	i.v	p.o	i.v	p.o	i.v	p.o
		3 mg/kg	30 mg/kg	3 mg/kg	30 mg/kg	3 mg/kg	30 mg/kg	3 mg/kg	30 mg/kg
Physical appearance	Post-dose	Normal	Normal	Normal	Normal	Normal	Normal	Normal	Normal
BUN (mg/dL)	Post-dose	16.00 ± 2.00	12.00 ± 1.73	12.83 ± 2.64	12.17 ± 2.86	14.17 ± 1.72	14.00 ± 2.00	14.17 ± 1.33	9.83 ± 1.47*
Creatinine (mg/dL)	Post-dose	0.63 ± 0.21	0.75 ± 0.08	0.73 ± 0.04	0.66 ± 0.03	0.73 ± 0.02	0.71 ± 0.02	0.70 ± 0.03	0.75 ± 0.02
AST (U/L)	Post-dose	69.21 ± 6.53	56.67 ± 2.08	65.83 ± 19.66	60.50 ± 7.12	59.50 ± 5.61	63.83 ± 11.77	64.17 ± 11.05	53.83 ± 6.88
ALT (U/L)	Post-dose	33.67 ± 10.07	17.33 ± 3.06	20.33 ± 2.42*	21.23 ± 2.44	18.83 ± 3.92*	22.50 ± 5.89	28.50 ± 5.82	19.33 ± 2.50
ALP (U/L)	Post-dose	60.33 ± 13.32	39.67 ± 5.86	44.67 ± 7.09	32.67 ± 7.45	49.50 ± 14.46	39.17 ± 11.20	48.50 ± 14.00	35.83 ± 8.38

AST: aspartate transaminase; ALT: alanine transaminase; ALP: alkaline phosphatase; BUN: blood urea nitrogen.

Data are expressed as mean ± SD, (n = 6).

*p < 0.05 for significant differences between control and treatment.

### 3.2 Pharmacokinetics

**FIGURE 1:**
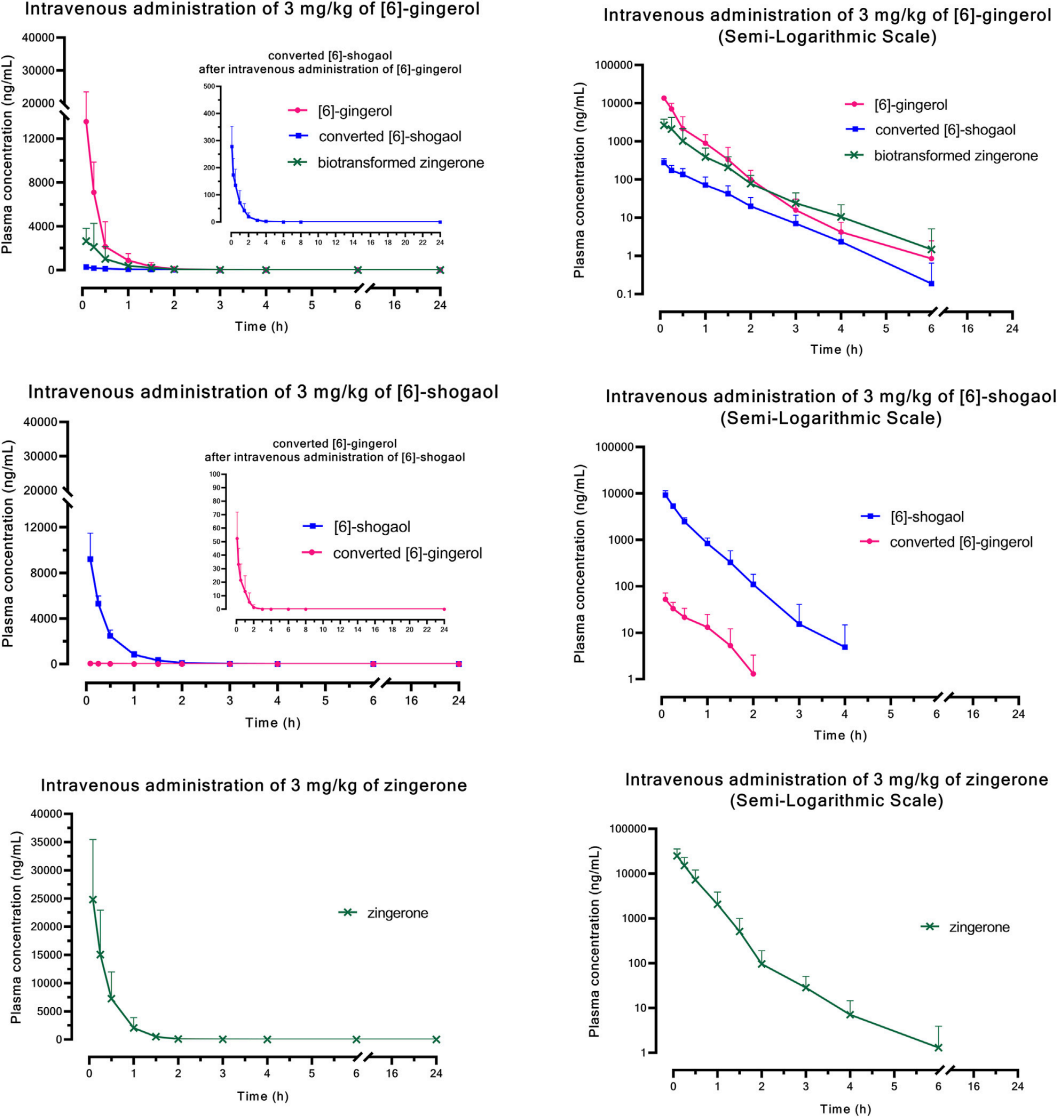
Comparative mean plasma concentration *versus* time profiles after a single intravenous administration of [6]-gingerol, [6]-shogaol, or zingerone. Data are presented as means ± SD (n = 6), linear scale and semi-logarithmic scale.

**FIGURE 2:**
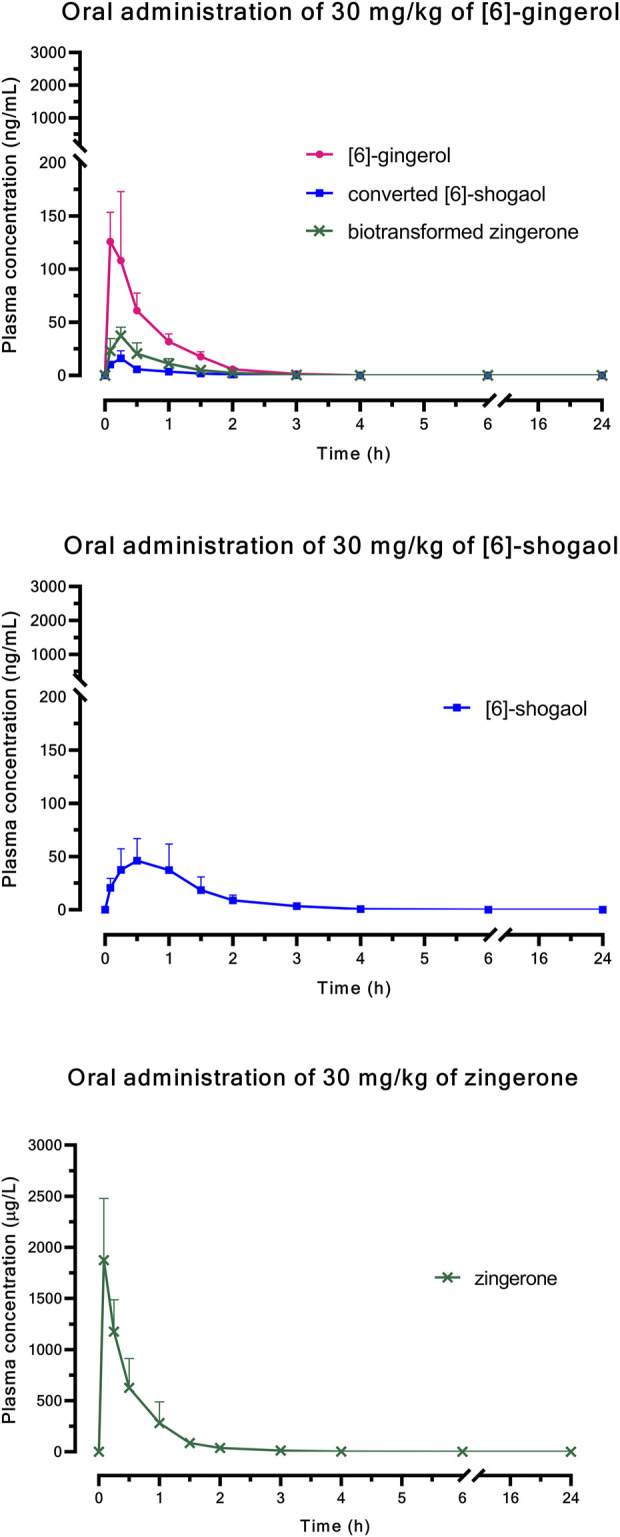
Comparative mean plasma concentration *versus* time profiles after a single oral administration of [6]-gingerol, [6]-shogaol, or zingerone. Data are presented as means ± SD (n = 6).

Figure 1 (i.v.) and Figure 2 (p.o.) illustrate the comparative mean plasma concentration-time profiles of 6G, 6S, and zingerone, while Tables 2 (i.v.) and 3 (p.o.) detail their corresponding pharmacokinetic parameters. Following intravenous (3 mg/kg) or oral (30 mg/kg) administration of pure compounds, the unchanged form of zingerone was observed in plasma at higher concentrations compared to both 6G and 6S. Zingerone’s AUC_(0–24 h)_ values were consistently higher than the corresponding values for 6G and 6S (AUC zingerone > 6G > 6S), regardless of administration route. The C_0_ (concentration at 0 min) values aligned with the AUC values (C_0_ zingerone > 6G > 6S) after intravenous administration of each compound. The parent compounds of these substances (6G, 6S, and zingerone) demonstrated rapid absorption, reaching peak plasma concentration (C_max_) within 5–45 min following oral administration. Their absolute oral bioavailability remained restricted at less than 2%, ranging from 0.10% to 0.40% and 0.10%–0.31% for 6G and 6S, respectively while zingerone showed higher values ranging from 0.32% to 1.60%.

**TABLE 2:** Pharmacokinetic parameters after a single intravenous administration of [6]-gingerol, [6]-shogaol and zingerone in rats.

PK parameters		[6]-gingerol intravenous administration (n = 6)						
		[6]- gingerol	converted [6]-shogaol		zingerone		[6]-gingerol glucuronide	
C_0_ ^a^	ng/mL	18,942.13 ± 7,401.41	1,846.82 ± 1,250.66		4,218.79 ± 1,346.52		N/A	
AUC_(0-t)_ ^a^	ng-h/mL	5,403.25 ± 2,028.16	280.40 ± 90.06		1,681.23 ± 1,028.50		11,087.06 ± 7,432.61	
AUC_(0-∞)_ ^a^	ng-h/mL	5,403.64 ± 2,027.91	281.89 ± 89.16		1,690.34 ± 1,033.02		11,087.09 ± 7,432.598	

PK parameters		[6]-shogaol intravenous administration (n = 6)						
		[6]-shogaol	converted [6]-gingerol		zingerone		[6]-shogaol glucuronide	
C_0_ ^a^	ng/mL	11,347.27 ± 1,826.87	238.03 ± 255.12		125.01 ± 81.16		N/A	
AUC_(0-t)_ ^a^	ng-h/mL	4,390.23 ± 853.32	41.39 ± 22.54		16.45 ± 7.23		7,966.49 ± 3,782.71	
AUC_(0-∞)_ ^a^	ng-h/mL	4,390.82 ± 853.12	42.38 ± 22.72		18.10 ± 5.49		7,974.90 ± 3,786.32	

PK parameters		zingerone intravenous administration (n = 6)						
		zingerone					zingerone glucuronide	
C_0_ ^a^	ng/mL	35,145.58 ± 15,745.78					N/A	
AUC_(0-t)_ ^a^	ng-h/mL	12,630.52 ± 5,515.28					12,873.76 ± 12,580.54	
AUC_(0-∞)_ ^a^	ng-h/mL	12,636.40 ± 5,512.57					12,903.70 ± 12,585.11	

^**a**^ Data are expressed as mean ± SD (n = 6).

Abbreviations: AUC, area under the plasma concentration–time curve.

**TABLE 3:** Pharmacokinetic parameters after a single oral administration of [6]-gingerol, [6]-shogaol and zingerone in rats.

PK parameters		[6]-gingerol oral administration (n = 6)				
		[6]- gingerol	converted [6]-shogaol		zingerone	[6]-gingerol glucuronide
C_max (obs)_ ^a^	ng/mL	144.94 ± 52.97	16.77 ± 6.31		40.49 ± 4.99	539.42 ± 377.66
T_max (obs)_ ^b^	h	0.08 [0.00]	0.25 [0.13]		0.25 [0.00]	0.25 [0.00]
AUC_(0-t)_ ^a^	ng-h/mL	92.11 ± 26.88	10.16 ± 4.01		28.03 ± 7.40	549.22 ± 272.42
AUC_(0-∞)_ ^a^	ng-h/mL	92.11 ± 26.88	10.35 ± 4.14		28.03 ± 7.40	603.05 ± 268.67
%Oral bioavailability^a^		0.17 ± 0.11				
		(0.10%–0.40%)				

PK parameters		[6]-shogaol oral administration (n = 6)				
		[6]-shogaol	converted [6]-gingerol		zingerone	[6]-shogaol glucuronide
C_max (obs)_ ^a^	ng/mL	51.79 ± 20.95	N/A		N/A	18.54 ± 6.93
T_max (obs)_ ^b^	h	0.75 [0.69]	N/A		N/A	0.375 [0.25]
AUC_(0-t)_ ^a^	ng-h/mL	66.91 ± 30.52	N/A		N/A	11.88 ± 1.87
AUC_(0-∞)_ ^a^	ng-h/mL	66.91 ± 30.52	N/A		N/A	11.89 ± 1.87
%Oral bioavailability^a^		0.15 ± 0.10				
		(0.10%–0.31%)				

PK parameters		zingerone oral administration (n = 6)				
		zingerone				zingerone glucuronide
C_max (obs)_ ^a^	ng/mL	1874.50 ± 603.81				4593.37 ± 1192.68
T_max (obs)_ ^b^	h	0.08 [0.00]				0.25 [0.00]
AUC_(0-t)_ ^a^	ng-h/mL	943.82 ± 348.33				5847.24 ± 134.65
AUC_(0-∞)_ ^a^	ng-h/mL	950.42 ± 372.98				5847.24 ± 134.65
%Oral bioavailability^a^		0.75 ± 0.52				
		(0.32%–1.60%)				

^a^ Data are expressed as mean ± SD, (n = 6).

^b^ Data are expressed as median [IQR].

Abbreviations: C_max_, maximum plasma concentration; T_max_, time to reach maximum concentration; AUC, area under the plasma concentration–time curve.

### 3.3 Interconversion

**FIGURE 3:**
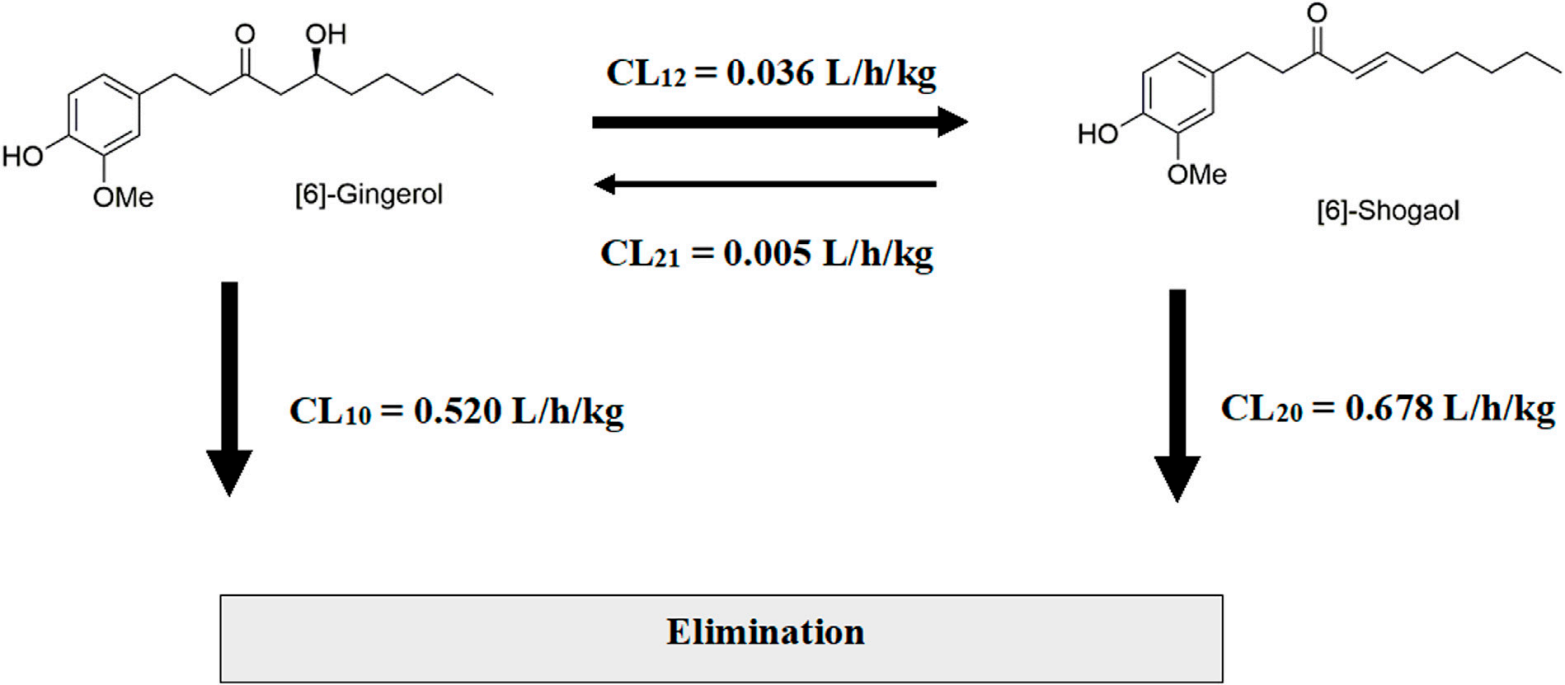
Interconversion of [6]-gingerol and [6]-shogaol after a single intravenous administration in rats.

Considering the *in vivo* interconversion between 6G and 6S, the concentrations of the administered parent compounds were higher than those of their respective converted compounds. Following intravenous administration of 6G, the AUC_(0–24 h)_ for its converted product (a converted 6S) constituted 5% of the AUC_(0–24 h)_ for its unchanged 6G throughout the 24-h period (Table 2). Notably, the conversion of 6G to 6S was more efficient than the reverse conversion (6S → 6G), with the AUC_(0–24 h)_ for converted 6G accounting for only 1% of the unchanged 6S. Figure 3 illustrates the interconversion clearance (L/h/kg) between 6G and 6S after intravenous administration in rats. The clearance of 6G (CL_12_) was approximately sevenfold greater than the reverse clearance (CL_21_, 6S → 6G). Both 6G and 6S also exhibited irreversible metabolic clearances (CL_10_ and CL_20_, respectively) that were significantly higher than their reversible clearances (CL_12_ and CL_21_). Consistent with the observation from an intravenous route, an oral administration of 6G also exhibited conversion to 6S. However, the conversion of 6S to 6G after oral administration was minimal, with undetectable levels of its converted product (Table 3).

### 3.4 Biotransformation

**FIGURE 4:**
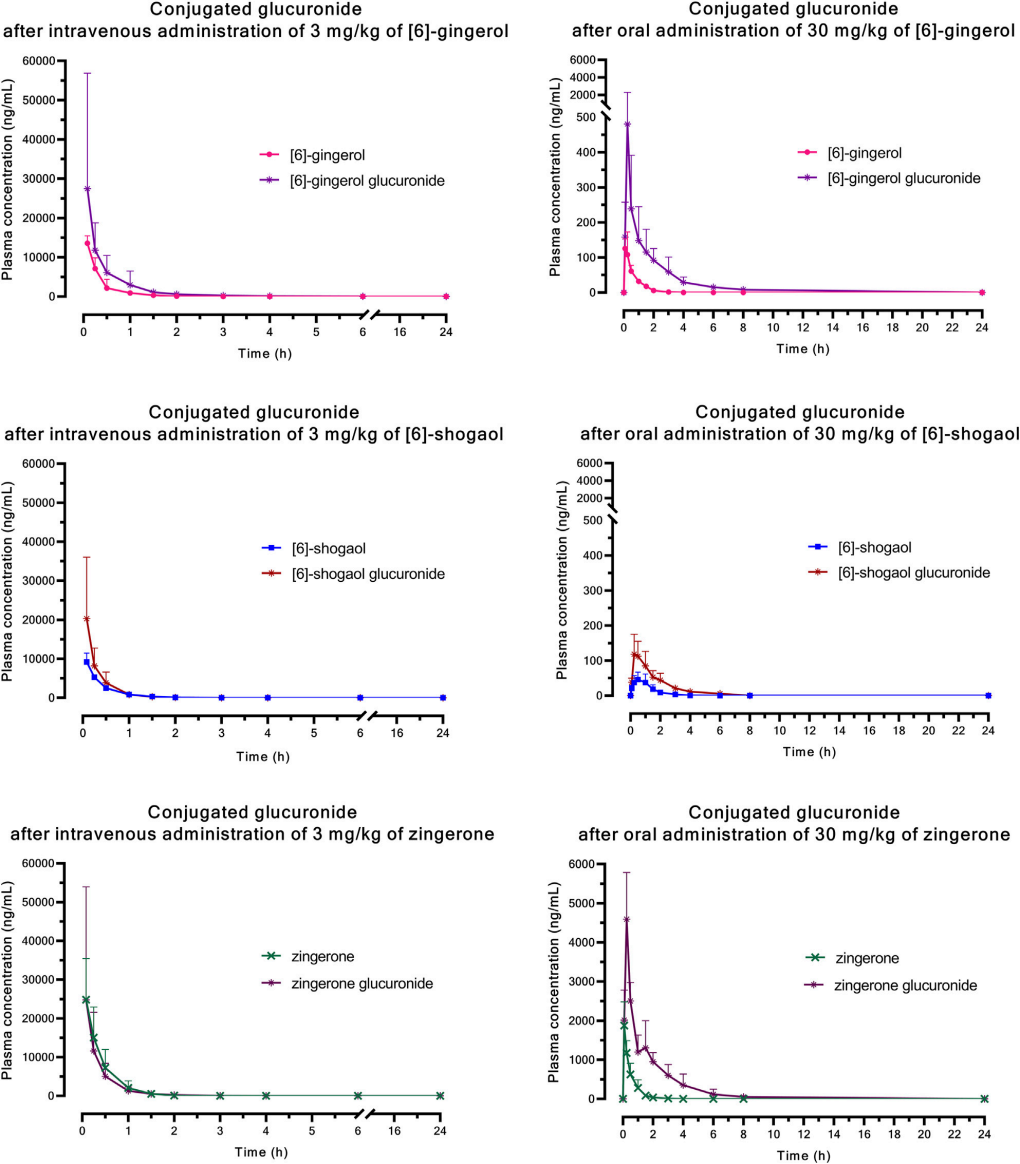
Mean plasma concentration *versus* time profiles of glucuronide conjugation after a single intravenous or oral administration of [6]-gingerol, [6]-shogaol, or zingerone in rats. Data are presented as means ± SD (n = 6).

The significantly higher plasma concentrations of glucuronide conjugates of 6G, 6S, and zingerone compared to their respective parent compounds constitute evidence of their possible metabolism in the Phase II metabolic pathway. These pure compounds exhibited a rapid appearance of conjugated metabolites, detectable within 15 min in plasma levels. The AUC_(0–∞)_ of the zingerone glucuronide following zingerone oral administration (Table 3) showed a 6-fold increase compared to the AUC_(0–∞)_ of the unconjugated zingerone. This AUC_(0–∞)_ of the zingerone glucuronide was remarkably higher compared to those of the other glucuronide metabolites measured after administering 6G or 6S (Table 3, Figure 4).

In this study, the potentially irreversible conversion among 6G and 6S by measuring plasma levels of zingerone after administering either 6G or 6S, was also investigated. The results showed that zingerone was detectable in plasma even after individual rats received 6G without zingerone, strongly suggesting a metabolic pathway in which 6G undergoes structural cleavage to retain its core structure as zingerone.

### 3.5 Tissue distribution and elimination

**FIGURE 5:**
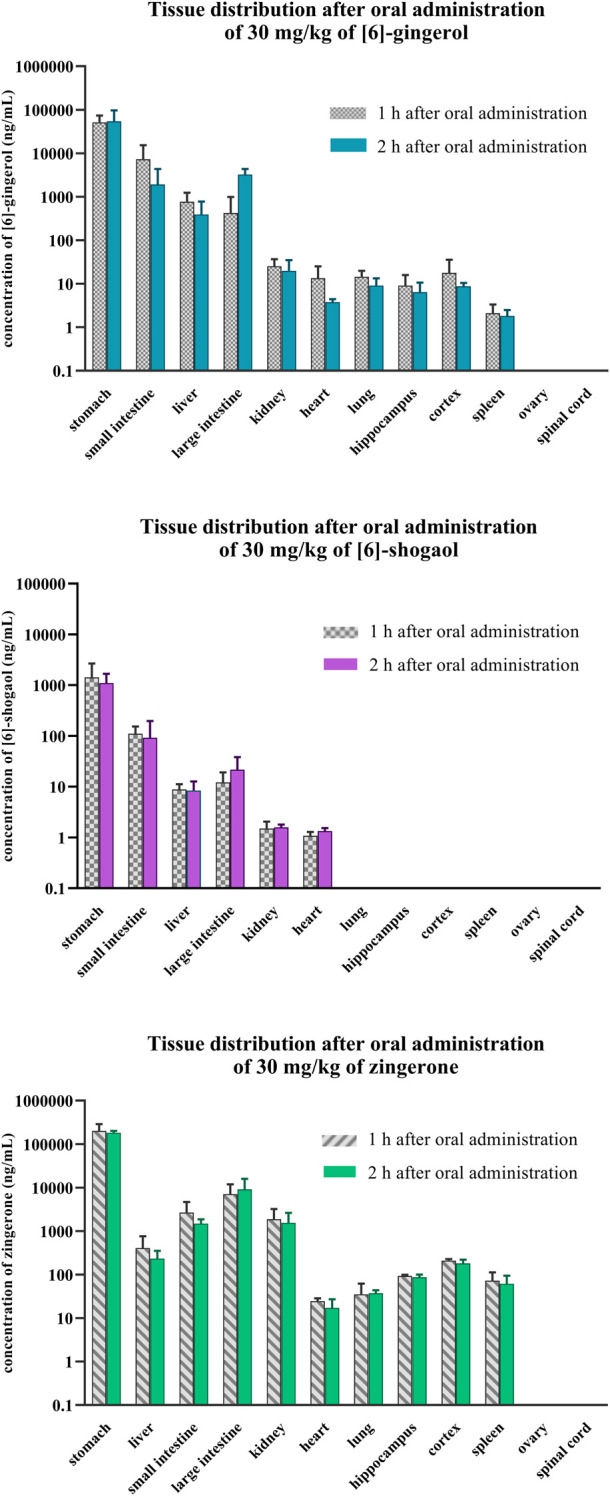
Tissue distribution after a single oral administration of [6]-gingerol, [6]-shogaol, and zingerone in rats. Data are presented as means ± SD (n = 3).

Within 1–2 h after oral administration, the presence of 6G, 6S, and zingerone in several organs demonstrated their wide distribution (Figure 5, Supplementary Figures S3). They primarily concentrated in the digestive organs (stomach, small intestine, and large intestine), liver, and kidney. Zingerone and 6G exhibited higher concentrations in the pharmacologically relevant brain regions of the hippocampus and cortex; however, 6S showed limited distribution, partially localizing to the digestive organs and lacking presence in key brain regions. Zingerone had the highest tissue-to-plasma ratios, followed by 6G and then 6S (Supplementary Figure S3). The stomach exhibited the highest ratios for all related compounds, suggesting potential site-specific biological activities. A higher tissue-to-plasma ratio indicated greater accumulation within the organs.

The parent compounds of 6G, 6S, and zingerone underwent minimal renal excretion, with less than 1% of each compound recovered in urine during 24–48 h. A negligible amount (<0.1%) of these unchanged forms was observed in fecal excretion within 48 h after intravenous or oral administration. The conjugated metabolites of 6G, 6S, and zingerone were excreted through the kidneys with an almost 10% recovery compared to the administered doses (Table 4).

**TABLE 4:** Percent recovery in urine and feces after intravenous or oral administration of [6]-gingerol, [6]-shogaol and zingerone in rats.

Elimination	% Recovery excretion					
	Parent compounds			Conjugated glucuronide metabolites		
	[6]-gingerol	[6]-shogaol	zingerone	[6]-gingerol glucuronide	[6]-shogaol glucuronide	zingerone glucuronide
Intravenous administration						
Urine						
T 0–24 h	0.623 ± 0.706	0.003 ± 0.002	0.184 ± 0.286	5.712 ± 2.768	0.153 ± 0.112	7.396 ± 6.349
T 24–48 h	0.008 ± 0.005	<0.001	<0.001	0.146 ± 0.073	<0.001	<0.001
Feces						
T 0–24 h	0.005 ± 0.002	<0.001	0.049 ± 0.027	N/A	0.003 ± 0.003	N/A
T 24–48 h	0.005 ± 0.001	N/A	0.002 ± 0.001	N/A	0.004 ± 0.001	N/A
Oral administration						
Urine						
T 0–24 h	0.100 ± 0.102	0.001 ± 0.001	0.049 ± 0.032	3.416 ± 1.735	0.042 ± 0.037	4.245 ±3.009
T 24–48 h	0.002 ± 0.001	<0.001	<0.001	0.036 ± 0.027	<0.001	0.166 ± 0.075
Feces						
T 0–24 h	0.001 ± 0.001	0.001 ± 0.001	0.004 ± 0.003	N/A	N/A	N/A
T 24–48 h	<0.001	0.002 ± 0.002	0.057 ± 0.017	N/A	N/A	N/A

%Recovery excretion represents the percentage of the administered dose of the test compound that is recovered in the excreta (urine and feces) over a specific time period (24 or 48 h).

## 4 Discussion

### 4.1 Safety and tolerability

Single intravenous (3 mg/kg) or oral (30 mg/kg) administration of 6G, 6S, or zingerone demonstrated favorable tolerability in rats, as evidenced by their physiological parameters and physical appearances, as well as analyses of liver enzymes and kidney function biomarkers in plasma. The administration of intravenous 6G or 6S significantly decreased ALT levels, while oral zingerone significantly lowered BUN levels compared to the control group (20% v/v DMSO). These observations suggest potential improvements in liver and kidney functions, respectively, and warrant further investigation to confirm the safety and potential benefits of these ginger constituents in liver and kidney deficit functions.

### 4.2 Pharmacokinetics

Although previous research has reported the limited oral bioavailability for ginger constituents (Li et al., 2019; Zhang et al., 2022), this study is the first study to provide a precise value on the individual oral bioavailability of 6G, 6S, and zingerone, which is generally below 2%. These low values can be attributed to several factors. First, the lipophilic nature of these compounds hinders optimal absorption in the water-rich intestinal environment (Bakht et al., 2014). Additionally, a significant portion of each compound undergoes extensive first-pass metabolism in the liver before reaching systemic circulation (Mukkavilli et al., 2017). Furthermore, efflux transporters likely contribute to reduced bioavailability by actively pumping these compounds back into the intestinal lumen (Kim T. H. et al., 2018). Finally, the interconversion between 6G and 6S can further influence their overall bioavailability.

Oral administration of pure 6G or zingerone demonstrated rapid absorption, reaching detectable plasma levels within 5 min. 6S achieved peak plasma concentrations within 45 min. This rapid uptake was likely due to the small size of their molecular structures (<300 g/mol), facilitating their rapid entry into blood circulation. The rapid absorption observed in the current study is consistent with the previous findings in the literature. Xu et al. (2016) and Jiang et al. (2008) also reported a T_max_ of 10 min for 6G after oral administration in rats. Intravenous administration resulted in a rapid decline in plasma concentrations of the parent compounds within 2 h, as observed in a prior study demonstrating rapid clearance of free 6G (Ding et al., 1991). This finding likely results from the intravenous route bypassing first-pass metabolism, leading to a significantly faster entry into the blood circulation and a shorter residence in systemic exposure compared to oral administration (Wang et al., 2017; Li et al., 2019).

The comparative pharmacokinetics investigated in this study illustrated the dominance of zingerone, as evidenced by its substantially greater AUC compared to both 6G and 6S after intravenous or oral administration of each pure compound. Although zingerone is present in minimal amounts in ginger or ginger extract, it emerges as one of the most prominently detected compounds in systemic circulation and in target organs. The higher bioavailability of zingerone and 6G, as reflected in their significantly higher tissue distribution compared to 6S (Figure 5, Supplementary Figures S3), suggested that they may accumulate in target organs and exert prolonged systemic effects. The presence of these compounds may contribute to the therapeutic properties of ginger and its observed health benefits.

### 4.3 Interconversion

The assessment of interconversion between 6G and 6S relied on AUCs obtained from the intravenous route to avoid potential confounding factors associated with the oral route, specifically first-pass metabolism and the influence of diverse enzymes and microbiota in the gastrointestinal tract. These factors can significantly impact the interpretation of compound conversion. Therefore, the study focused on clearance values associated with the interconversion process as observed through the intravenous route. The *in vivo* interconversion findings indicated a preferential conversion of 6G to 6S compared to the reverse process. This conversion is potentially facilitated by the labile *β*-hydroxy ketone group in the molecular structure of 6G, which readily undergoes dehydration to form the *α*,*β* conjugated ketone 6S (Bhattarai et al., 2001; Bhattarai et al., 2007; Kou et al., 2017). This latter structure (6S) gains additional stability from the conjugated carbonyl system. On the other hand, the negligible conversion of 6S to 6G after oral administration of 6S, resulted in undetectable or below-quantifiable levels of its converted product (converted 6G) in plasma throughout the 24-h period. This observation warrants further investigation into a broader range of oral 6S dosages to elucidate potential dose-dependent effects on interconversion pharmacokinetics, particularly the impact of first-pass metabolism and specific enzymatic pathways involved in the unidirectional conversion. The irreversible elimination clearance (CL_10_ and CL_20_) exhibited higher clearance in comparison to those observed in the interconversion clearance (CL_12_ and CL_21_).

Our comprehensive understanding of the *in vivo* interconversion process clarifies the previously unexplained discrepancy between the administered dosage and the observed plasma levels of 6G and 6S. A study by Li et al. (2019) that investigated the pharmacokinetics of an orally administered ginger extract in rats described a significant discrepancy in the plasma levels of these two key compounds. Although the rats received an extract with a higher concentration of 6G, the observed plasma levels of 6G were lower than those of 6S. A more in-depth understanding of the interconversion between 6G and 6S, as explored in this current study, provides an explanation. A portion of the ingested 6G undergoes conversion to 6S, and some of the 6G is also metabolized through glucuronide conjugation. Meanwhile, the reverse transformation of 6S to become 6G is negligible, suggesting that 6S may persist in the systemic circulation after the oral administration of the ginger extract. The total amount of 6S in the systemic circulation includes both the directly administered 6S parent compound and the portion converted from 6G when orally administered as an extract.

**FIGURE 6:**
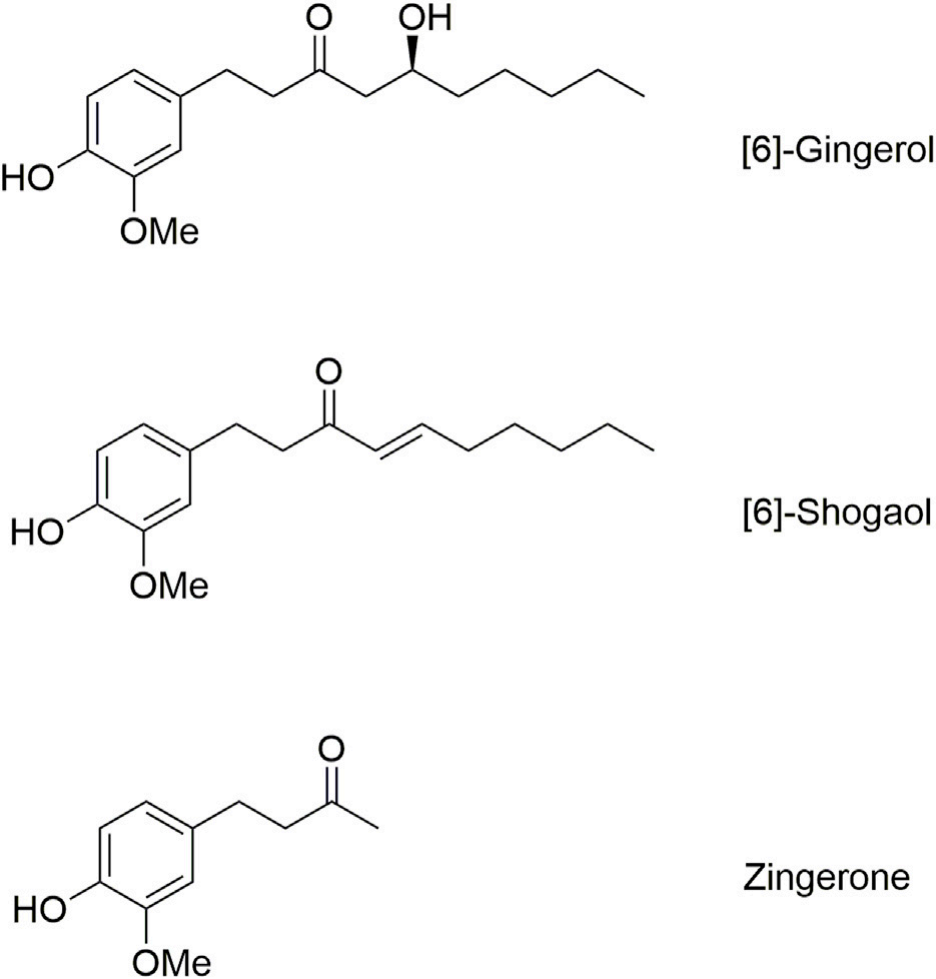
Molecular structures of [6]-gingerol, [6]-shogaol, and zingerone.

The structure of zingerone is present in both 6G and 6S as the shared core structure of these ginger compounds. As shown in Figure 6, 6G and 6S feature this core structure along with a straight-chain alkane structure with a hydroxy group or a double bond in its carbon chain. This study demonstrated measurable levels of zingerone after the intravenous or oral administration of 6G. The finding probably results from the biotransformation of 6G, wherein its *β*-hydroxy ketone side chain is cleaved through the retro-aldol reaction, resulting in the core structure being transformed into zingerone. Conversely, zingerone was not detected after 6S administration, potentially due to the low absorption of 6S leading to minimal biotransformation of 6S into zingerone, or potentially due to alternative metabolic pathways of 6S.

### 4.4 Biotransformation

Following oral administration, parent compounds of 6G, 6S, and zingerone demonstrated an extended presence in plasma for up to 4 h compared to the intravenous route. A study by Zick et al. (2008) showed that ginger compounds are rapidly absorbed after oral dosing and are eliminated as glucuronide conjugates, with [6]-gingerol-4′-*O*-*β*-glucuronide being detected in the bile. Another study suggested glucuronidation as one of the metabolic pathways for 6G, 6S, and zingerone (Li et al., 2019). It can be inferred that the observed irreversible biotransformation partially proceeds through glucuronidation, followed by biliary excretion of these conjugated metabolites. Our findings revealed the persistence of conjugated metabolites of 6G, 6S, and zingerone in the blood circulation for up to 8 h after oral administration, indicating prolonged systemic exposure. Zingerone stands out with a significantly higher AUC_(0–∞)_ for its glucuronide. In contrast, 6S exhibited a markedly lower AUC for its glucuronide, indicating other alternative metabolic pathways. This finding is consistent with a previous study demonstrating 6S’s ability to be metabolized into the potent anti-inflammatory compound, [6]-paradol (Tokuhara et al., 2013).

### 4.5 Tissue distribution and elimination

This study revealed that zingerone exhibited a higher tissue distribution compared to both 6G and 6S, likely due to its smaller size (molecular weight of zingerone = 194.23 g/mol) facilitating easier penetration through cell membranes. The consistent tissue distribution observed between 1 and 2 h after oral administration suggested that the compounds remain concentrated within their target organs for at least 1–2 h. These findings support and extend previous observations of gingerol distribution in rats (Li et al., 2019). The compounds were widely distributed across various organs, with a notable accumulation in the GI tract compared to other tissues, implying that the GI system is the primary target of these bioactive ginger components. Significant evidence highlights ginger’s efficacy in alleviating gastrointestinal discomfort, particularly nausea, and vomiting; the accumulation of these compounds in the GI tract observed in this study aligns with the previous evidence (Nikkhah Bodagh et al., 2019). This current study also indicated a partial distribution of zingerone and 6G in the hippocampus and cortex, specific brain regions crucial for learning and memory, warranting further investigation to elucidate their potential cognitive benefits. Further inquiry into these compounds to explore their mechanisms of action, identify optimal dosages, and assess their long-term effects can significantly expand the evidence for ginger’s efficacious use in enhancing cognitive function or neuroprotection (Wattanathorn et al., 2011; Kim C. Y. et al., 2018).

Less than 1% of parent compounds were excreted unchanged in urine and feces within 48 h of administration. This finding highlights the limited systemic exposure of these compounds along with their potentially extensive tissue distribution or alternative elimination pathways. Glucuronidation enhances the compounds’ polarity, facilitating elimination, in part, through hepatic and renal excretion. Conjugated glucuronides are excreted within 24 h after oral administration, indicating the clearance of these metabolites.

### 4.6 Druggability of ginger active constituents: 6-gingerol, 6-shogaol, and zingerone

According to Lipinski’s rule, an orally active drug-like compound should have no more than one violation of the following four parameters: possess no more than 5 hydrogen bond donors, possess no more than 10 hydrogen bond acceptors, have a molecular weight below 500 Da, and have an octanol-water partition coefficient (log P) less than 5 (Lipinski, 2000). All selected active constituents of ginger (6G, 6S and zingerone) meet Lipinski’s criteria, indicating their drug-like attributes based on physicochemical characteristics.

The observed interconversion pharmacokinetics potentially hold conceptual significance for understanding the behavior of 6G and 6S. Given the multi-constituent nature of ginger, a comprehensive understanding of its several major active components is crucial for its development as a traditional herbal medicine. This study highlights the reversible conversion of 6G and 6S, suggesting the importance of considering interconversion between these two major bioactive constituents when establishing therapeutic doses for ginger. This knowledge can provide fundamental insights for dose optimization by accounting for the degree of 6G and 6S interconversion in dose calculations.

## 5 Conclusion

The administration of a single intravenous dose (3 mg/kg) or oral dose (30 mg/kg) of either 6G, 6S, or zingerone was demonstrated to be well-tolerated in rats. These parent compounds exhibited rapid absorption; however, their oral bioavailability remained notably restricted (<2%) due to their limited systemic circulation, extensive tissue distribution, and substantial biotransformation. The conversion of 6G to 6S demonstrated a larger clearance than the reverse conversion (6S → 6G). The irreversible biotransformation partially involves phase II glucuronide conjugation. The parent compounds and their respective conjugated metabolites were subsequently excreted in urine, with negligible elimination in feces. Integrating interconversion pharmacokinetics into dosage design is imperative for precise adjustments in clinical applications, thereby maximizing the synergistic therapeutic efficacy of orally administered combined ginger constituents (6G, 6S, and zingerone) and minimizing their potential for adverse events in humans.

## Data Availability

The raw data supporting the conclusion of this article will be made available by the authors, without undue reservation.

## Abbreviations

AAALAC, American Association for Accreditation of Laboratory Animal Care; ALP, Alkaline phosphatase; ALT, Alanine transaminase; ANOVA, Analysis of variance; AST, Aspartate transaminase; AUC, Area under plasma concentration-time curve; BUN, Blood urea nitrogen; CE, Collision energy; Cmax, Maximum plasma concentration; CL, Clearance; CV, Coefficient of variation; DMSO, dimethyl sulfoxide; ESI, Electrospray ionization; GI tract, Gastrointestinal tract; HPLC, High performance liquid chromatography; IACUC, Institutional Animal Care and Use Committee; IQR, Interquartile range; IS, Internal standard; i.v., Intravenous administration; LC-MS/MS, Liquid chromatography-tandem mass spectrometry; LLOQ, Lower limit of quantification; MRM, Multiple reaction monitoring; p.o., Oral administration; PVDF membrane, Polyvinylidene fluoride membrane; QC sample, Quality control sample; RSD, Relative standard deviation; SD, Standard deviation; Tmax, Time to reach maximum plasma concentration; 6G, [6]-Gingerol; 6S, [6]-Shogaol.
