# Supplementary material for: Unraveling the interconversion pharmacokinetics and oral bioavailability of the major ginger constituents: [6]-gingerol, [6]-shogaol, and zingerone after single-dose administration in rats
Source: Front Pharmacol. 2024 Jun 6;15:1391019. doi: 10.3389/fphar.2024.1391019 (PMC11187260; doi:10.3389/fphar.2024.1391019)
Supplement: Supplementary file 1 [file DataSheet1.pdf]

Supplementary Material

Unraveling the interconversion pharmacokinetics and oral bioavailability of the major ginger constituents: [6]-gingerol, [6]-shogaol, and zingerone after single-dose administration in rats

Phanit Songvut[a,†], Watanyoo Nakareangrit[b,†], Wanida Cholpraipimolrat[a], Jackapun Kwangjai[c], Luksamee Worasuttayangkurn[a], Piyajit Watcharasit[a,d], Jutamaad Satayavivad[a,d*]

[a] Laboratory of Pharmacology, Chulabhorn Research Institute, Bangkok, Thailand

[b] Translational Research Unit, Chulabhorn Research Institute, Bangkok, Thailand

[c] Food and Drug Quality Unit, Chulabhorn Research Institute, Bangkok, Thailand

[d] Center of Excellence on Environmental Health and Toxicology (EHT), OPS, MHESI, Thailand

[†] Equally contributing authors

*** Correspondence:**

Assoc. Prof. Jutamaad Satayavivad, Ph.D.

E-mail address: jutamaad@cri.or.th

Table S1. Intra- and inter-day accuracy and precision of [6]-gingerol, [6]-shogaol and zingerone in rats' plasma, urine and feces

[6]-gingerol concentration (μg/L)		Plasma		Urine		Feces	
		Accuracy (%)	CV (%)	Accuracy (%)	CV (%)	Accuracy (%)	CV (%)
**Intra-day**							
**HQC**	**2000.00**	99.63	1.37	99.62	3.67	102.52	8.38
**MQC**	**1000.00**	99.58	4.18	101.10	5.08	104.30	7.34
**LQC**	**7.81**	107.13	3.68	108.32	6.61	106.88	6.55
**LLOQ**	**3.91**	113.49	3.81	109.55	4.95	112.15	5.84
**Inter-day**							
**HQC**	**2000.00**	99.96	1.11	95.84	3.59	102.16	1.45
**MQC**	**1000.00**	100.79	1.07	101.12	1.56	98.98	5.22
**LQC**	**7.81**	107.06	0.94	102.03	6.43	105.00	1.94
**LLOQ**	**3.91**	110.86	2.29	103.51	5.27	110.93	1.12

[6]-shogaol concentration (μg/L)		Plasma		Urine		Feces	
		Accuracy (%)	CV (%)	Accuracy (%)	CV (%)	Accuracy (%)	CV (%)
**Intra-day**							
**HQC**	**2000.00**	99.27	1.81	104.15	4.95	102.01	5.81
**MQC**	**1000.00**	102.64	5.32	93.25	2.56	95.17	5.96
**LQC**	**7.81**	99.85	5.85	98.93	2.22	90.45	2.66
**LLOQ**	**3.91**	88.36	7.59	105.62	4.35	110.15	6.70
**Inter-day**							
**HQC**	**2000.00**	99.89	1.00	97.03	6.79	101.57	1.49
**MQC**	**1000.00**	103.39	1.18	90.94	2.20	96.65	4.66
**LQC**	**7.81**	98.44	4.43	101.42	2.30	97.82	7.58
**LLOQ**	**3.91**	89.20	1.46	99.14	6.14	106.78	2.74

zingerone concentration (μg/L)		Plasma		Urine		Feces	
		Accuracy (%)	CV (%)	Accuracy (%)	CV (%)	Accuracy (%)	CV (%)
**Intra-day**							
**HQC**	**2000.00**	99.69	1.01	99.29	2.60	98.58	2.80
**MQC**	**1000.00**	99.30	2.98	95.34	4.24	94.78	6.06
**LQC**	**7.81**	105.45	8.51	102.16	7.88	105.71	4.66
**LLOQ**	**3.91**	106.19	13.46	96.98	8.24	94.28	9.17
**Inter-day**							
**HQC**	**2000.00**	102.12	3.49	102.29	4.78	101.35	2.89
**MQC**	**1000.00**	100.33	2.43	95.55	1.65	98.87	8.24
**LQC**	**7.81**	103.50	4.19	103.15	1.25	99.51	7.51
**LLOQ**	**3.91**	105.99	1.07	97.11	1.29	98.85	6.05

lower limit of quantitation (LLOQ), low quality control concentration (LQC concentration), medium quality control concentration (MQC concentration), high quality control concentration (HQC concentration)

Table S2. Stability of [6]-gingerol, [6]-shogaol and zingerone in rats' plasma, urine and feces under different storage conditions

	Storage conditions	Concentration (µg/L)	[6]-gingerol		[6]-shogaol		zingerone	
			%Accuracy	%CV	%Accuracy	%CV	%Accuracy	%CV
Plasma	3 freeze-thaw cycles	2000.00 (HQC)	92.90	4.38	93.69	1.96	95.45	2.18
		1000.00 (MQC)	91.24	4.33	96.20	5.38	89.23	4.01
		7.81 (LQC)	96.87	5.52	94.96	3.73	93.81	7.67
	2 months at -40 °C	2000.00 (HQC)	91.82	4.36	95.22	2.38	92.72	5.19
		1000.00 (MQC)	92.18	3.11	96.21	2.23	93.12	4.34
		7.81 (LQC)	96.90	6.89	91.96	5.89	91.72	4.02
	24 h at autosampler	2000.00 (HQC)	92.93	5.52	90.70	4.10	95.76	2.45
		1000.00 (MQC)	92.15	3.41	95.73	5.63	92.85	5.50
		7.81 (LQC)	100.83	3.73	93.36	6.77	96.17	7.44
Urine	3 freeze-thaw cycles	2000.00 (HQC)	96.78	4.22	94.99	2.05	91.05	5.31
		1000.00 (MQC)	91.31	3.12	88.97	2.25	87.17	2.74
		7.81 (LQC)	94.71	6.87	89.47	1.53	93.53	4.92
	2 months at -40 °C	2000.00 (HQC)	95.82	2.70	91.48	3.88	94.36	0.94
		1000.00 (MQC)	92.00	4.24	88.81	2.07	92.23	5.26
		7.81 (LQC)	94.20	3.73	91.29	5.45	91.73	4.82
	24 h at autosampler	2000.00 (HQC)	94.33	5.02	92.60	5.67	90.03	4.77
		1000.00 (MQC)	91.17	3.93	89.27	4.71	87.77	2.44
		7.81 (LQC)	94.96	7.37	93.98	2.67	95.92	6.81
Feces	3 freeze-thaw cycles	2000.00 (HQC)	92.82	5.54	95.55	3.63	92.17	2.71
		1000.00 (MQC)	92.09	6.51	92.45	7.12	92.32	6.72
		7.81 (LQC)	91.82	4.56	87.69	4.55	96.66	5.37
	2 months at -40 °C	2000.00 (HQC)	96.92	6.07	93.99	4.47	92.84	5.11
		1000.00 (MQC)	88.92	1.35	88.10	2.47	92.27	2.47
		7.81 (LQC)	93.75	7.47	86.24	1.78	96.75	6.50
	24 h at autosampler	2000.00 (HQC)	91.82	7.59	93.51	5.43	89.10	3.23
		1000.00 (MQC)	90.68	5.03	92.68	5.49	92.86	6.17
		7.81 (LQC)	93.58	4.94	87.68	1.49	95.86	6.44

low quality control concentration (LQC concentration), medium quality control concentration (MQC concentration), high quality control concentration (HQC concentration)

Table S3. Notation for dose and AUC of Interconversion

Administered compounds	Measured area	Dose	AUC
gingerol	gingerol	$\mathrm{Dose}^{\mathrm{gingerol}}$	$\mathrm{AUC}_{\mathrm{gingerol}}^{\mathrm{gingerol}}$
gingerol	shogaol	$\mathrm{Dose}^{\mathrm{gingerol}}$	$\mathrm{AUC}_{\mathrm{shogaol}}^{\mathrm{gingerol}}$
shogaol	gingerol	$\mathrm{Dose}^{\mathrm{shogaol}}$	$\mathrm{AUC}_{\mathrm{gingerol}}^{\mathrm{shogaol}}$
shogaol	shogaol	$\mathrm{Dose}^{\mathrm{shogaol}}$	$\mathrm{AUC}_{\mathrm{shogaol}}^{\mathrm{shogaol}}$

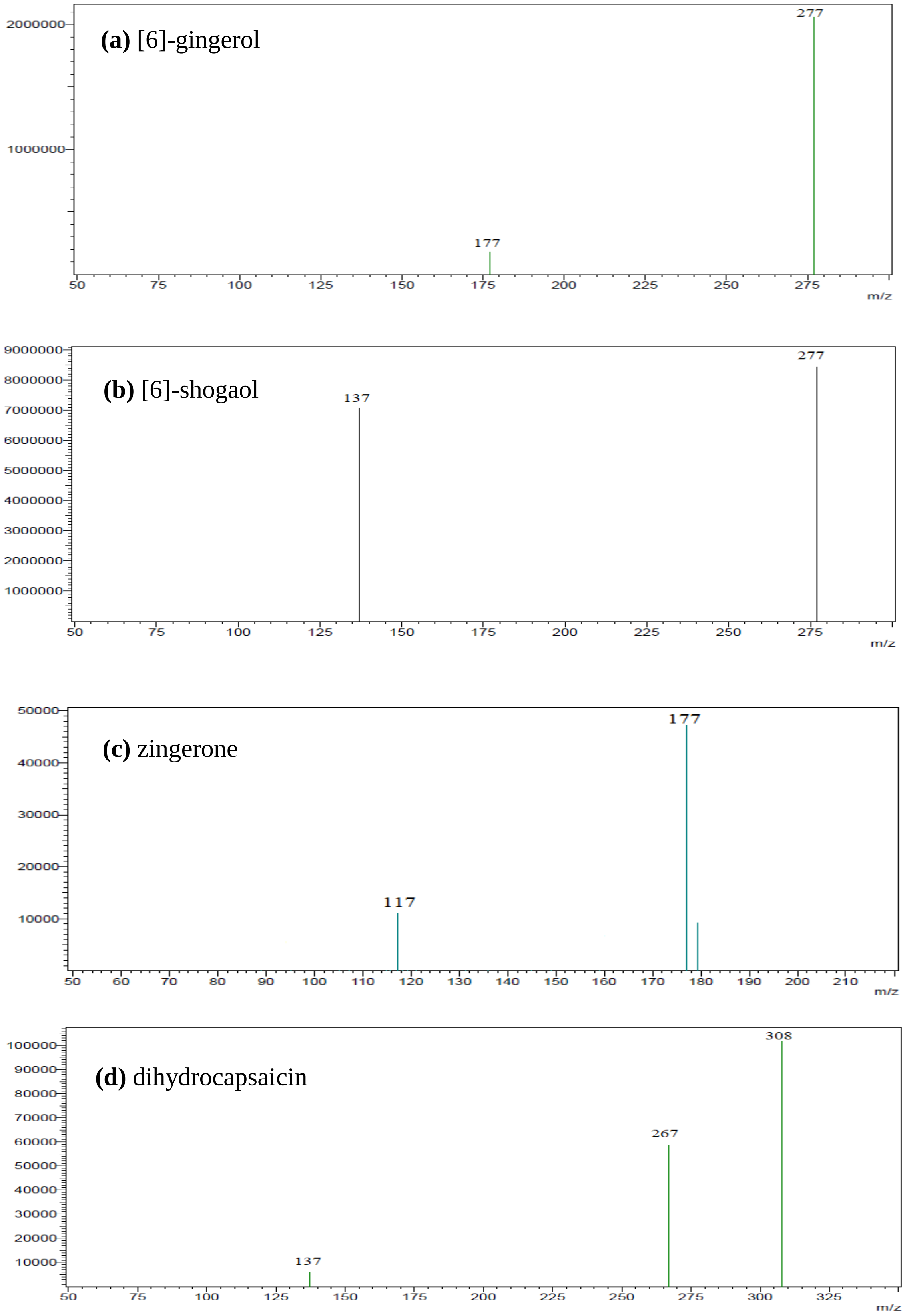

Figure S1. Mass spectrums of **(a)** [6]-gingerol, **(b)** [6]-shogaol, **(c)** zingerone, and **(d)** dihydrocapsaicin (internal standard, IS)

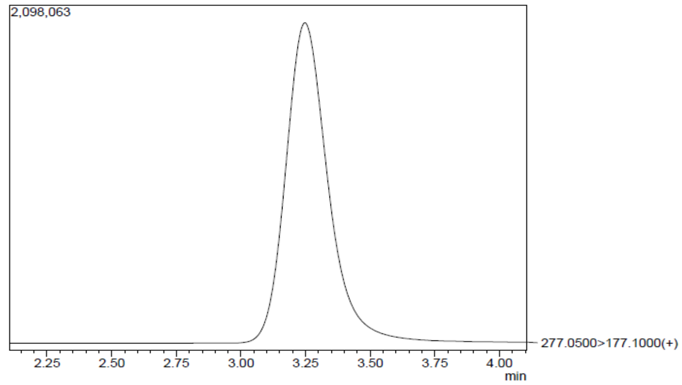

(a) [6]-gingerol

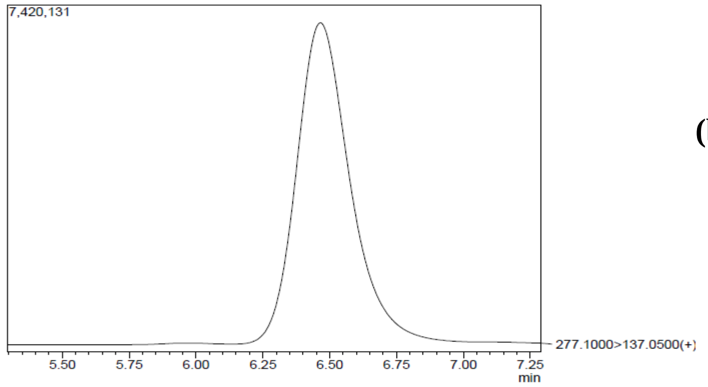

(b) [6]-shogaol

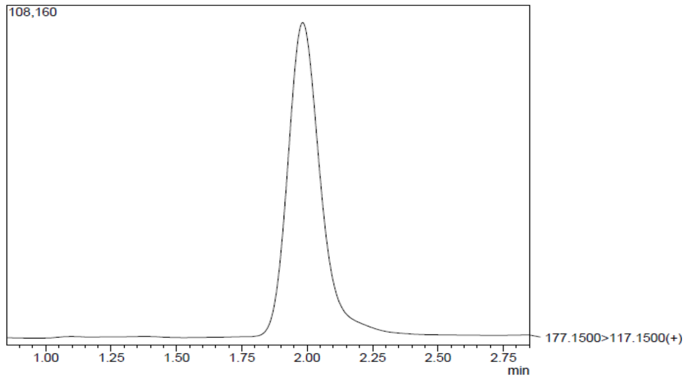

(c) zingerone

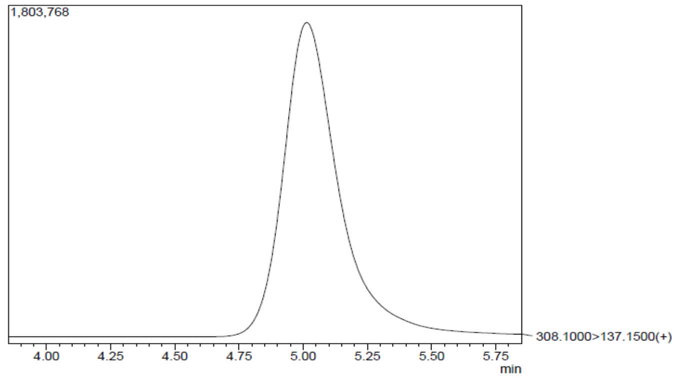

(d) dihydrocapsaicin

Figure S2. MS Chromatograms of **(a)** [6]-gingerol, **(b)** [6]-shogaol, **(c)** zingerone, and **(d)** dihydrocapsaicin (internal standard, IS)

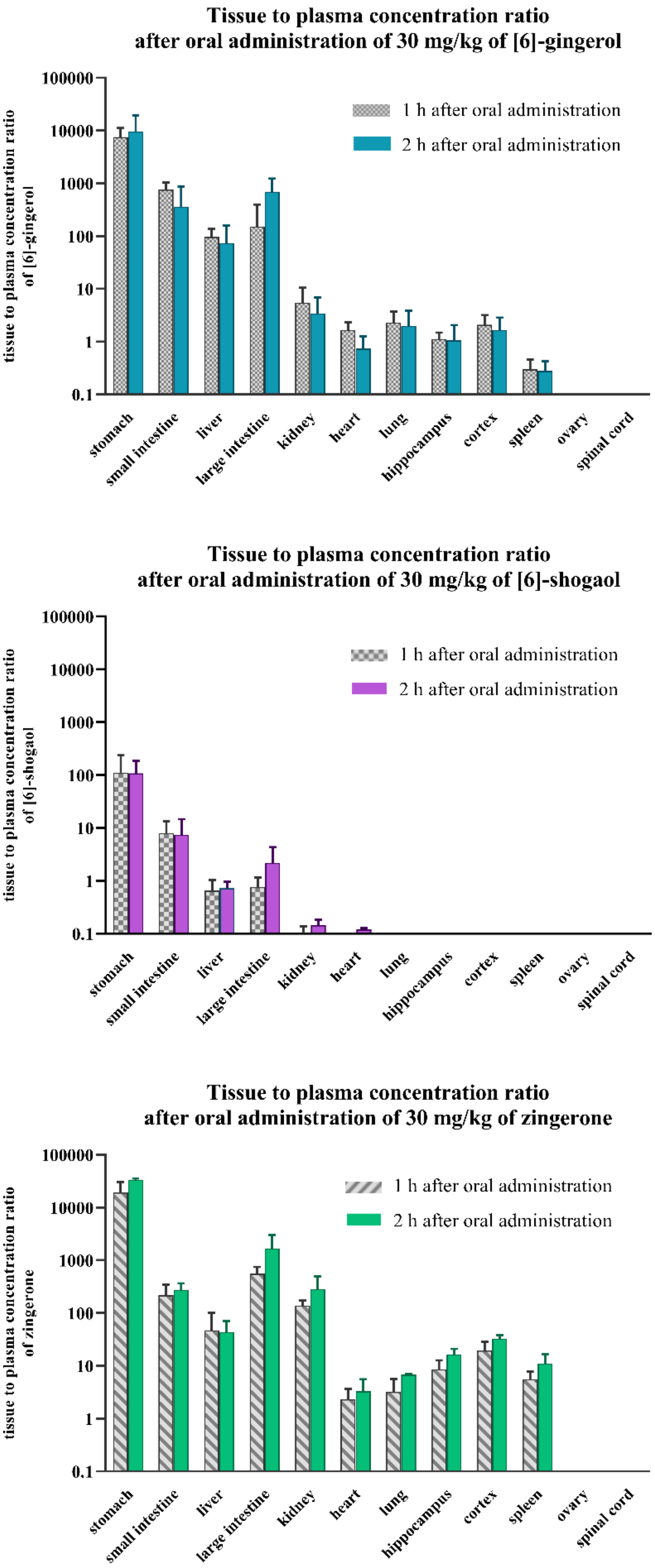

Figure S3. Tissue to plasma concentration ratio after a single oral administration of [6]-gingerol, [6]-shogaol, and zingerone in rats. Data are presented as means ± SD (n=3)
